# A response to “Response to a Trial on Reversal of Death by Neurologic Criteria”

**DOI:** 10.1186/s13054-017-1614-4

**Published:** 2017-02-12

**Authors:** Ira S. Pastor

**Affiliations:** Bioquark Inc., BNY Mellon Center, 1735 Market St, #3750, Philadelphia, PA 19103 USA

The author is grateful for the opportunity to write this rebuttal to Dr. Ariane Lewis and Dr. Arthur Caplan’s “Response to a Trial on Reversal of Death by Neurologic Criteria”.

While the time Dr. Lewis and Dr. Caplan took to write is appreciated, their critique ignores key learnings from both recent biomedical history and parallel research disciplines within the life sciences, and shows intransigence in an era of novel clinical development models, emerging legislative initiatives regarding no-option patients, and regulatory advantages brought about by the globalization of health care.

Living cadaver research has been pursued legally and ethically in many countries, including the USA (http://articles.baltimoresun.com/2003-02-02/news/0302020371_1_research-living-dead-university-of-pittsburgh), for years now, to utilize the human model in toxicology, pharmacokinetic, and pharmacodynamic studies, as well as for the testing of novel surgical techniques and medical devices.

Since the early days of regenerative biology, it has been well known that a range of nonhuman organisms can repair, regenerate, and remodel substantial portions of their brain and brain stem even after critical life-threatening trauma, including certain amphibians (http://onlinelibrary.wiley.com/doi/10.1111/j.1440-169X.2007.00914.x/pdf), fish, planarians, metamorphic insects, and small hibernating mammals.

There have been dozens of reported cases (http://www.ncbi.nlm.nih.gov/pubmed/19818943) in the literature violating the irreversibility label of the 1968 Harvard ad-hoc brain death criteria, primarily in young subjects who most likely retain some neurogenic niche. Although controversial and resulting in poor outcomes, such cases highlight that things are not always black or white in our understanding of the severe disorders of consciousness.

Epimorphosis is a highly complex form of regeneration involving many synergistic mechanisms (http://onlinelibrary.wiley.com/doi/10.1002/dvdy.22553/pdf), including but not limited to cellular reprogramming, targeted histolysis, and modulation of the innate immune response. In spite of the comfort that the flawed, reductionist drug development model brings to the evidence-based medicine dynamic, any “single magic bullet” approach to recapitulate such dynamics would be fairly futile, and instead requires creative thinking, especially in an era of personalization of medicine, adaptive clinical trial design, and ongoing legislative initiatives for experimental therapy access for “living” patients.

“False hope” is unfortunately created by a global medical establishment that the public sees generating $7 trillion annually, yet provides no cures for most of the chronic degenerative diseases responsible for human suffering and death. Exploratory research programs of this nature are not false hope. They are a glimmer of hope.

